# Immunity to *Lutzomyia whitmani* Saliva Protects against Experimental *Leishmania braziliensis* Infection

**DOI:** 10.1371/journal.pntd.0005078

**Published:** 2016-11-03

**Authors:** Regis Gomes, Katrine Cavalcanti, Clarissa Teixeira, Augusto M. Carvalho, Paulo S. Mattos, Juqueline R. Cristal, Aline C. Muniz, José Carlos Miranda, Camila I. de Oliveira, Aldina Barral

**Affiliations:** 1 Escritório Técnico Regional, Fundação Oswaldo Cruz (FIOCRUZ), Teresina, Piauí, Brazil; 2 Instituto Gonçalo Moniz, Fundação Oswaldo Cruz (FIOCRUZ), Salvador, Bahia, Brazil; 3 Federal University of Bahia School of Medicine, Salvador, Brazil; 4 INCT - Instituto de Investigação em Imunologia, São Paulo, Brazil; 5 Universidade Federal da Bahia, Salvador, Brazil; Queensland Institute of Medical Research, AUSTRALIA

## Abstract

**Background:**

Previous works showed that immunization with saliva from *Lutzomyia intermedia*, a vector of *Leishmania braziliensis*, does not protect against experimental infection. However, *L*. *braziliensis* is also transmitted by *Lutzomyia whitmani*, a sand fly species closely related to *Lu*. *intermedia*. Herein we describe the immune response following immunization with *Lu*. *whitmani* saliva and the outcome of this response after *L*. *braziliensis* infection.

**Methods and findings:**

BALB/c mice immunized with *Lu*. *whitmani* saliva developed robust humoral and cellular immune responses, the latter characterized by an intense cellular infiltrate and production of IFN-γ and IL-10, by both CD4^+^ and CD8^+^ cells. Mice immunized as above and challenged with *L*. *braziliensis* plus *Lu*. *whitmani* saliva displayed significantly smaller lesions and parasite load at the challenge site. This protection was associated with a higher (p<0.05) IFN-γ production in response to SLA stimulation. Long-term persisting immunity was also detected in mice immunized with *Lu*. *whitmani* saliva. Furthermore, individuals residing in an endemic area for cutaneous leishmaniasis (CL) presented antibody responses to *Lu*. *whitmani* saliva. However CL patients, with active lesions, displayed a lower humoral response to *Lu*. *whitmani* saliva compared to individuals with subclinical *Leishmania* infection.

**Conclusion:**

Pre-exposure to *Lu*. *whitmani* saliva induces protection against *L*. *braziliensis* in a murine model. We also show that *Lu*. *whitmani* salivary proteins are immunogenic in naturally exposed individuals. Our results reinforce the importance of investigating the immunomodulatory effect of saliva from different species of closely related sand flies.

## Author Summary

The saliva from sand flies contains biologically active proteins that permit the insect to obtain a blood meal. When vertebrates are continuously exposed to these molecules, through insect biting, for example, they induce an immune response (antibody and cell-mediated immunity) in the vertebrate host. Previously, we showed that immunity to salivary proteins from *Lutzomyia intermedia* a vector of *Leishmania braziliensis*, the main species that causes cutaneous leishmaniasis (CL) in Brazil, did not protect but exacerbated CL. In the present work, we investigated if immunity to *Lutzomyia whitmani*, another vector of *L*. *braziliensis*, induced a similar effect or not. We observed that mice immunized with *Lu*. *whitmani* saliva develop immunity to the salivary components and that this immunity protected the mice against CL development. We further observed that people residing in areas where *Lu*. *whitmani* occurs also develop antibodies to saliva and that CL patients have lower levels of these antibodies. These evidences point to differences in the protein repertoire present in the saliva of different sand flies and highlight the concept that salivary proteins should be considered as additional components of a vaccine for leishmaniasis.

## Introduction

Leishmaniasis develops when an infected sand fly bites the skin of the mammalian host, injecting saliva and the infective forms of *Leishmania*. Sand fly saliva includes molecules that modulate the host’s hemostatic, inflammatory and immune responses creating a favorable environment that facilitate parasite infection (rev. in [1,2]). Experimental co-inoculation of sand fly saliva and *Leishmania sp*. resulted in significant lesion exacerbation and increased parasite load [3–6]. On the other hand, mice exposed to bites of uninfected sand flies [7,8] or immunized with specific salivary molecules [9,10] were protected against *Leishmania* infection. In these studies, protection against disease correlated with an intense recruitment of lymphocytes and macrophages to the infection site as well as production of IFN-γ and IL-12. The current hypothesis is that at the time of parasite inoculation, saliva presence recalls anti-saliva memory cells in previously exposed individuals and IFN-γ-producing cells promote *Leishmania* killing (rev. in [11]).

*Lutzomyia intermedia* and *Lutzomyia whitmani* are the main vectors responsible for *Leishmania braziliensis* transmission in Latin America [12] and *L*. *braziliensis* is the principal etiological agent of Cutaneous Leishmaniasis (CL) in Brazil. Different from other studies, immunity to *Lu*. *intermedia* salivary proteins did not prevent *L*. *braziliensis* infection and immunized mice developed more severe lesions that lasted longer [13,14]. CL patients living in an endemic area for *L*. *braziliensis* showed a higher anti-*Lu*. *intermedia* saliva IgG response compared to individuals displaying a subclinical infection [13] and exposure to *Lu*. *intermedia* saliva increases the risk of developing CL [15]. In the present work, we focused on characterizing the immune response to *Lu*. *whitmani* saliva since it is closely related to *Lu*. *intermedia* (both from the Nyssomyia subgenus) and may coexist in CL endemic areas [16]. We show that the immune response to *Lu*. *whitmani* saliva, characterized by the presence of antibodies and of IFN-γ-producing cells, protects against experimental CL development and that it is persistent. We also show that human CL patients have lower levels of anti- *Lu*. *whitmani* saliva compared to subjects with subclinical *L*. *braziliensis* infection.

## Methods

### Ethics statement

BALB/c mice (females), 6–8 weeks of age were obtained from the animal facility at IGM/FIOCRUZ and were maintained under pathogen-free conditions during experimentation. Animal work was conducted according to the Guidelines for Animal Experimentation of the Conselho Nacional de Controle de Experimentação Animal (CONCEA). The IGM-FIOCRUZ Ethics Committee on Animal Care and Utilization (CEUA) approved all procedures involving animals (CEUA-017/2013-IGM/FIOCRUZ). Research with human subjects was performed following approval of the Ethical Committee (CEP) of the Hospital Prof. Edgard Santos (Salvador, Bahia, Brazil, 240/2009) and CONEP (Brazil). A written informed consent was obtained from each participant. No minors participated in this study.

### Sand flies and preparation of SGS

*Lu*. *whitmani* sand flies were captured in Corte de Pedra (Bahia, northeastern Brazil). First, *Lu*. *whitmani* sand flies were morphologically identified according to the identification key proposed by Young and Duncan and females were dissected to obtain salivary glands that were kept at -70°C. Immediately before use, Salivary Glands (SGs) were disrupted by ultrasonication in 1.5-ml conical tubes and then centrifuged at 10,000 × g for two minutes. The supernatant was collected and used for the experiments. SGS preparations were tested for LPS (lipopolysaccharide) contamination using a commercially available LAL chromogenic kit (QCL-1000; Lonza Biologics) and LPS concentrations were < 0.1 ng/ml.

### Intradermal immunization with *Lu*. *whitmani* SGS

BALB/c mice were immunized three times, intradermally (ear), with *Lu*. *whitmani* SGS (equivalent to 1 pair of SGs) in 10μl of PBS or with PBS alone (control mice), using a 27G needle.

### Analysis of anti-saliva antibodies in mice

Anti-saliva antibody detection was performed as described [13]. ELISA plates (Nunc Maxisorp) were coated with 100 μl of SGS equivalent to 1 pair of SGS/ml in coating buffer (NaHCO_3_ 0.45 M, Na_2_CO_3_ 0.02 M, pH 9.6), overnight at 4°C. After washing with PBS-Tween, wells were blocked with PBS-Tween 0.05% plus 5% dried skim milk for 2h at room temperature. Wells were incubated with either pre-immune sera (obtained before immunizations) or with immune sera (obtained two weeks after each immunization) diluted (1:50) in PBS-Tween for 1 hour at 37°C. After further washings, wells were incubated with alkaline phosphatase-conjugated anti-mouse IgG antibody (Promega) diluted (1:1000) in PBS-Tween for one hour at 37°C. The reaction was developed with p-nitrophenylphosphate in sodium carbonate buffer pH9.6 with 1 mg/ml of MgCl_2_, after a final washing cycle. Absorbance was recorded at 405 nm.

### Western blot analysis

The equivalent of 10 pairs of SGs per lane were run in a 4–12% NuPAGE gels (Invitrogen) and stained by Coomassie Blue. For Western Blot analysis, salivary proteins were then transferred from NuPAGE gels to nitrocellulose membranes using the iBlot system (Invitrogen). Blocking of membranes was performed with 5% non-fat milk in PBS-0.05% Tween, pH 8.0, overnight at 4°C. Following blocking, membranes were incubated with immune sera (pooled from 5 immunized mice) obtained two weeks after the last immunization, diluted (1:20) in PBS-Tween. After further washings, membranes were incubated with diluted anti-mouse IgG alkaline phosphate-conjugated antibody (1:1000) in PBS-Tween for one hour at room temperature. The membrane was developed using an alkaline phosphatase substrate (Promega) and the reaction was stopped by washing with deionized water.

### Delayed-type hypersensitivity response

Following three intradermal immunizations with SGS (equivalent to 1 pair of SGs) or PBS (control mice) in the right ear dermis, mice were inoculated in the contra-lateral ear with *Lu*. *whitmani* SGS (equivalent to 1 pair of SGs). Forty-eight hours later, ear thickness was measured to determine induration using a digital caliper (Thomas Scientific). Mice were euthanized and the ear tissue removed and fixed in 10% formaldehyde. Following fixation, tissues were processed, embedded in paraffin, and 5-μm sections were stained with hematoxylin and eosin (H & E) for analysis.

### Flow Cytometry

Following three intradermal immunizations with SGS (equivalent to 1 pair of SGs) in the right ear dermis, mice were inoculated with *Lu*. *whitmani* SGS (equivalent to 1 pair of SGs) in the left ear dermis. Flow cytometry analysis of cytokine producing cells, present in draining lymph nodes, was performed as described [17]. Forty eight hours later, mice were euthanized and lymph node cells draining the challenge site were obtained and cultured (1x10^6^) in one mL of RPMI 1640 containing 10% FBS, L-glutamine and penicillin/streptomycin in flat-bottom 96-well plates at 37°C and 5% CO_2_ for 18h, in the presence of 10μg/ml of anti-CD3 (145-2C11) and anti-CD28 (37.51) (Ebioscience). Brefeldin A (BD GolgiPlug; BD Pharmingen) was added to all the wells during the last 4h of culture (1μl). Cells were then harvested and washed with PBS and blocked with anti-CD16/CD32 (BD Fc block, 2.4G2; BD Pharmingen) for 30 minutes at 4°C. Surface staining was performed with PerCP-labeled anti-CD4 (RM4-5), FITC-labeled anti-TCR-β (H57-597), all from BD Pharmingen, for 30 minutes. Cells were washed twice, fixed and permeabilized with Cytofix/Cytoperm Plus (BD Pharmingen) for 30 minutes at 4°C. Intracellular staining of cytokines was performed with PE-labeled anti-IFN-γ (XMG 1.2) and PE0Cy7-labeled anti-IL-4 (11B11) from BD Pharmingen, for 30 minutes. A minimum of 100,000 cells was acquired using a FACSAria flow cytometer (BD Biosciences). Data were analyzed using Flow Jo software.

### Detection of Cytokines by ELISA

Two weeks after the last immunization with *Lu*. *whitmani* SGS or with PBS (control mice), mice were euthanized and draining lymph node cells were cultured (1x10^6^) in RPMI medium containing 10% FBS, L-glutamine and penicillin/streptomycin in flat-bottom 48-well plates. Cells were stimulated with SGS (equivalent to 2 pairs of SGs) or with soluble *L*. *braziliensis* antigen (SLA, 50μg/ml). Supernatants were collected 72 hours after incubation to detect production of IFN-γ, IL-4 and IL-10 by ELISA (Ebiosciences).

### Intradermal challenge with SGS and *L*. *braziliensis* parasites

Two weeks after the last immunization, saliva-exposed mice were inoculated with live parasites plus saliva as described [13]. Briefly, mice were infected in the left ear dermis with stationary-phase *L*. *braziliensis* promastigotes (10^5^ parasites) + SGS (equivalent to 1 pair of SGs) in 10 μl of saline. Development of lesion was monitored weekly using a digital caliper (Thomas Scientific).

### Parasite load estimate

Parasite quantification was performed as described elsewhere [17]. Briefly, total ear and draining lymph node tissue were obtained and homogenized. Serial dilutions were performed (1:5) in 96-well flat bottom microtiter plates containing 200 μl Schneider's medium (Life Technologies) supplemented with 20% heat inactivated fetal bovine serum, 2 mM L-glutamine, 100 U/ml penicillin and 100μl/ml streptomycin. Parasite growth was monitored for 7 days of incubation at 26°C. The number of viable parasite in each ear tissue was estimated from the highest dilution at which *Leishmania* promastigotes could be grown.

### Human study population

A prospective cohort was established in January 2010 and was followed up to January 2013 [18]. Inclusion criteria for enrollment consisted of negative history of any type of *Leishmania* infection and absence of signs consistent with previous CL or ML such as scars on the skin or mucosal area, criteria fulfillment were established following a medical interview. Additionally, enrolled individuals co-inhabited the same household as CL patients, the latter diagnosed following parasite isolation or a positive PCR for *L*. *braziliensis*. This study included: i) patients with CL (n = 30), ii) subjects with subclinical *L*. *braziliensis* infection (n = 33) and iii) individuals with a negative history of *Leishmania* infection (n = 231). All participants were from Corte de Pedra (Bahia, Brazil), an area endemic for CL with high transmission of *L*. *braziliensis* [19]. The diagnosis of CL was confirmed by parasite isolation or a *L*. *braziliensis*-positive polymerase chain reaction result. Subclinical CL cases were defined as those presenting a positive *Leishmania* Skin Test (LST) without previous history of CL [20]. LST was performed using Soluble *Leishmania* Antigen prepared from *L*. *braziliensis*, as described [21]. Additionally, 72.7% of subclinical individuals presented positive serology to *L*. *braziliensis*.

### Analysis of anti-saliva antibodies in humans

Evaluation of anti-*Lu*. *whitmani* saliva antibodies was performed as described elsewhere [15]. ELISA microplates were coated 1 hour at 37°C and overnight at 4°C with 100 μl of SGS equivalent to 1 pair of SGS/ml in coating buffer (NaHCO_3_ 0.45 M, Na_2_CO_3_ 0.02 M, pH 9.6). After washing with PBS-Tween, blocking was performed with PBS-Tween plus 1% Bovine serum albumin (BSA) for 2 hours at RT. Wells were incubated for 1 hour with diluted sera (1:100) in PBS-Tween plus 0,25% of BSA. After further washings, wells were incubated with alkaline phosphatase conjugated anti-human IgG antibody (Promega) diluted (1:5000) in PBS-Tween plus 0,25% of BSA, for 1 hour at 37°C. Following a final washing cycle, wells were developed with p-nitrophenyl-phosphate in sodium carbonate buffer pH9.6 with 1 mg/ml of MgCl_2_. The plate was recorded at an absorbance at 405nm.

### Statistical analysis

GraphPad software (Prism V. 5.0) was used for all statistical analysis. A two-tailed unpaired Student’s t-test was used. Data from studies performed with human sera were analyzed by Mann-Whitney test for comparisons between 2 groups and those among 3 or more groups by Kruskal–Wallis test followed by Dunn multiple comparison tests. *p* values of 0.05 or less were considered significant.

## Results

### Immunization with *Lu*. *whitmani* salivary proteins induces humoral and cellular immunity in BALB/c mice

**Fig 1:**
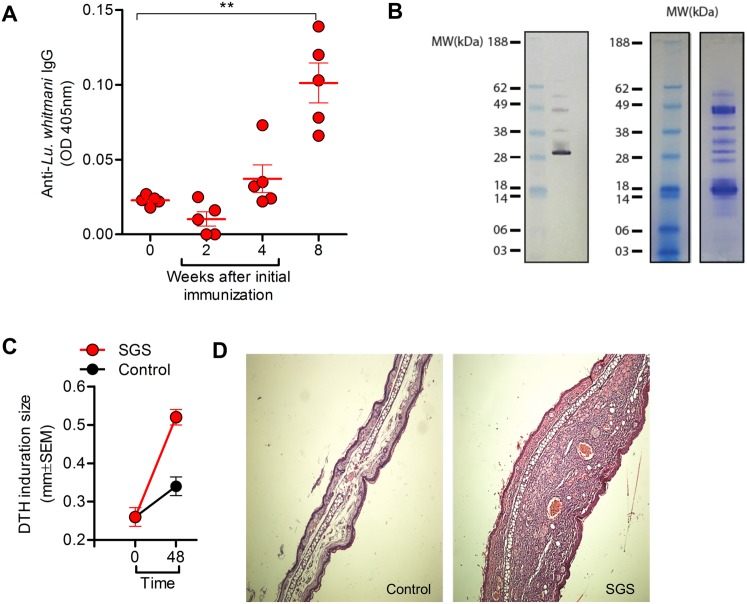
Immune response to *Lutzomyia whitmani* salivary molecules. BALB/c mice were immunized three times with *Lu*. *whitmani* SGS (equivalent to 1 pair of salivary glands) (red circles) or were inoculated with saline (control) (black circles), in the right ear, at two week intervals. (A) IgG respose to *Lu*. *whitmani* SGS determined by ELISA at different time points. Data are shown individually, from one representative experiment (bar at mean ± SEM). (B) Western blot analysis of *Lu*. *whitmani* salivary proteins using sera from immunized mice and SDS-PAGE depicting *Lu*. *whitmani* salivary proteins. (C) Two weeks after the last immunization, mice were challenged in the opposite ear with *Lu*. *whitmani* SGS and DTH response was measure 48h later. Data, shown as (median ± SD), are from two experiments performed with five mice in each group (D) Ear sections were obtained 48h later and stained with H&E. Sections were analyzed by optical microscopy under (100X). ** p<0.01.

To address whether salivary proteins from *Lu*. *whitmani* are immunogenic, we initially immunized mice three times with SGS and this resulted in the development of a humoral response, characterized by the presence of IgG (Fig 1A). Western blot analysis showed that the main proteins recognized by sera from *Lu*. *whitmani* SGS-immunized mice recognized antigens of ~38, ~30, ~36 and ~45 kDa (Fig 1B). We then evaluated whether a DTH reaction, a proxy for cellular immunity, developed in mice immunized with *Lu*. *whitmani* SGS as this effect has been shown for SGS from other sand fly species [3] and for defined salivary proteins [9]. Inoculation of SGS in the contra-lateral ear of immunized mice induced an increase in ear thickness, measured 48h after SGS inoculation (Fig 1C) and examination of ear sections also evidenced a strong cellular recruitment at the site of SGS inoculation (Fig 1D).

**Fig 2:**
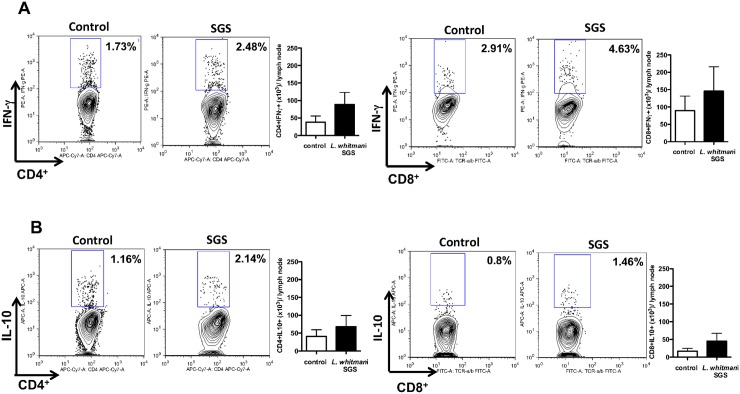
Frequency and absolute number of cytokine-producing cells in mice immunized with *Lutzomyia whitmani* SGS. BALB/c mice were immunized three times with *Lu*. *whitmani* SGS (equivalent to 1 pair of salivary glands) or were inoculated with saline (control), in the right ear, at two week intervals. Forty eight hours after SGS inoculation in the left ear dermis, mice were euthanized and draining lymph node cells were stimulated with anti-CD3 and anti-CD28 for 16h. Cells were subsequently stained for determination of frequency and absolute numbers of (**A**) CD4^+^TCR^+^IFN-^+^ and CD8^+^TCR^+^IFN-^+^ and (**B**) CD4^+^TCR^+^IL-10^+^ and CD8^+^TCR^+^IL-10^+^ T cells. The numbers shown represent mean ± SEM from three independent experiments, each performed with three mice. Frequency plots are representative from one experiment.

**Fig 3:**
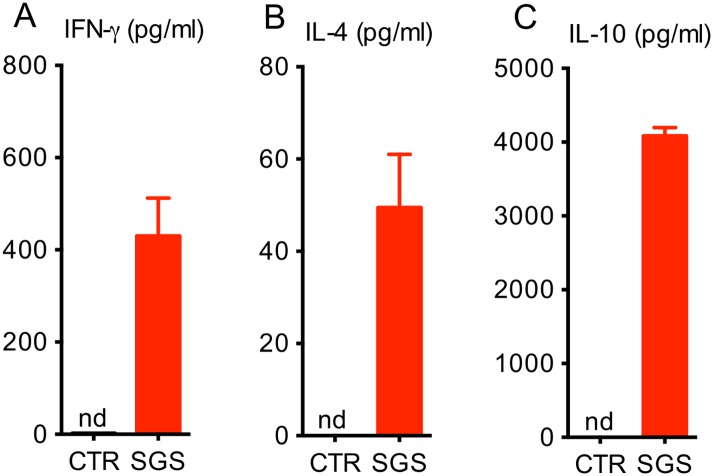
Cytokine production following immunization with *Lutzomyia whitmani* SGS. BALB/c mice were immunized three times with *Lu*. *whitmani* SGS (equivalent to 1 pair of salivary glands) or were inoculated with saline (control), in the right ear, at two week intervals. Two weeks after the last immunization mice were euthanized and draining lymph node cells were stimulated with *Lu*. *whitmani* SGS for 72h. Levels of (**A**) IFN-, (**B**) IL-4 or (**C**) IL-10 were determined in culture supernatants by ELISA. Data, shown as mean ± SEM, are from one representative experiment, performed with three mice in each group. nd, not detected.

Flow cytometric analysis of draining lymph node cells obtained from mice inoculated with *Lu*. *whitmani* SGS showed increased frequencies of cells expressing both IFN- (CD4^+^ and CD8^+^) (Fig 2A) and IL-10 (CD4^+^ and CD8^+^) (Fig 2B). Although the differences in the frequency of cytokine-positive cells were not significantly different, we observed a marked increase in IFN- (Fig 3A), IL-4 (Fig 3B) and IL-10 production (Fig 3C) following re-stimulation of draining lymph node cells with specific antigen (SGS), indicating the presence of a mixed cellular immune response.

### Immunization with *Lu*. *whitmani* SGS protects against *L*. *braziliensis* infection

**Fig 4:**
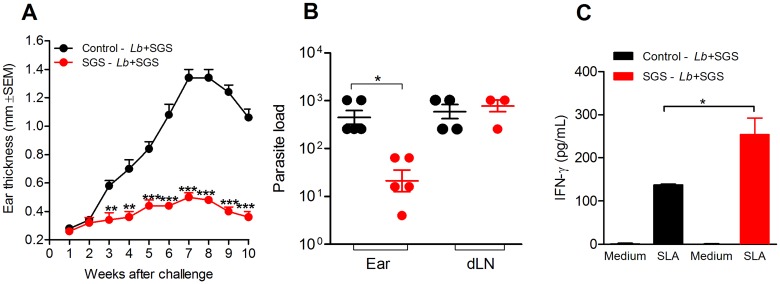
*Lutzomyia whitmani* saliva immunization induces protection against experimental *Leishmania braziliensis* infection. BALB/c mice were immunized three times with *Lu*. *whitmani* SGS (equivalent to 1 pair of salivary glands) (red circles) or were inoculated with saline (control) (black circles), in the right ear, at two week intervals. Two weeks after the last immunization mice challenged in the opposite ear with 10^5^ *L*. *braziliensis* plus SGS (equivalent to one pair of salivary glands). (**A**) Lesion development was monitored weekly. Data, shown as mean ± SEM, are from one representative experiment, performed with five mice in each group. (**B**) Parasite load was determined ten weeks after challenge via a limiting dilution assay. Data (bar at mean ± SEM) are shown individually. (**C**) Ten weeks after challenge, spleen cells were stimulated with Soluble *Leishmania* Antigen (SLA) and IFN- levels were measured in culture supernatants by ELISA, 72h later. Data, shown as mean ± SEM, are from one representative experiment, performed with five mice in each group. * p<0.05; **, p<0.01; ***, p< 0.001.

To test whether immunity to *Lu*. *whitmani* SGS would protect against CL, mice immunized with SGS were co-inoculated with *L*. *braziliensis* plus *Lu*. *whitmani* SGS. Following this challenge inoculation, immunized mice did not display lesions whereas control mice (immunized with PBS) showed an increase in ear thickness that progressed steadily until 7 weeks post challenge (Fig 4A). This absence of CL lesions in immunized mice correlated with a significantly lower parasite load at the challenge site (ear), measured 10 weeks later (Fig 4B). In draining lymph nodes, parasite load was similar comparing mice immunized with *Lu*. *whitmani* SGS versus controls. Cells obtained from immunized mice displayed a higher (p<0.05) production of IFN- following *in vitro* stimulation with soluble *Leishmania* antigen, compared to controls (Fig 4C). These results show that the immune response developed in mice immunized with *Lu*. *whitmani* SGS confers protection against a challenge infection with *L*. *braziliensis* plus SGS, inhibiting CL development. Protection was correlated with a dominant IFN- production in response to *Leishmania*.

**Fig 5:**
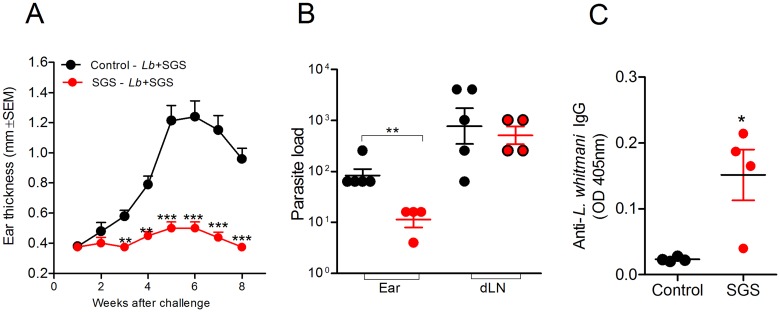
Persistent immunity induced by *Lutzomyia whitmani* saliva protects against experimental *Leishmania braziliensis* infection. BALB/c mice were immunized three times with *Lu*. *whitmani* SGS (equivalent to 1 pair of salivary glands) (red circles) or were inoculated with saline (control) (black circles), in the right ear, at two week intervals. Twelve weeks after the last immunization mice challenged in the opposite ear with 10^5^ *L*. *braziliensis* plus SGS (equivalent to one pair of salivary glands). (**A**) Lesion development was monitored weekly. Data, shown as mean ± SEM, are from one representative experiment, performed with five mice in each group. (**B**) Parasite load was determined eight weeks after challenge via a limiting dilution assay. Data (bar at mean ± SEM) are shown individually. (**C**) IgG response to *Lu*. *whitmani* SGS determined by ELISA at twelve weeks before challenge. Data are shown individually, from one representative experiment (bar at mean ± SEM). * p<0.05; **, p<0.01; ***, p< 0.001.

Previously, mice were immunized with *Lu*. *whitmani* SGS and were challenged two weeks after the last immunization (Fig 4). In a subsequent experiment, we immunized mice in the same scheme but challenged infection with *L*. *braziliensis* plus *Lu*. *whitmani* SGS occurred twelve weeks after the last immunization. Again, immunized mice failed to develop CL lesions (Fig 5A) and parasite load was also lower (p<0.05) at the challenge site (Fig 5B). These findings recapitulate results obtained when challenge was done two weeks after the last SGS immunization (Fig 4). IgG levels anti-*Lu*. *whitmani* SGS in protected mice were similar to those observed after the last immunization (Fig 1A), indicating that immunity persists for weeks after immunization.

### Humoral Immune Response to *Lu*. *whitmani* SGS in a CL endemic area

**Fig 6:**
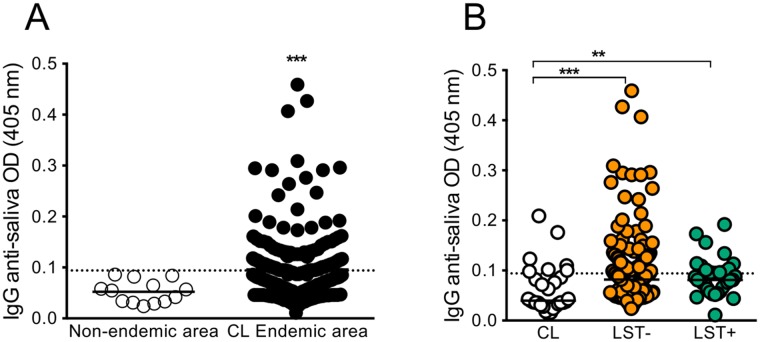
Human serum IgG response against *Lutzomyia whitmani* SGS. (**A**) ELISA was performed against *Lu*. *whitmani* SGS using human sera were from individuals from a Cutaneous Leishmaniasis (CL) endemic area (n = 264) or from control individuals (n = 13). (**B**) ELISA was performed against *Lu*. *whitmani* SGS with human sera from CL individuals (n = 30), sera from healthy individuals with either a negative (n = 231) or a positive (n = 33) *Leishmania* skin test (LST). The dotted line represents the cut-off value for the assay. Data are shown individually, bar at mean. p<0.01; ***, p< 0.001.

Having established that mice immunized with *Lu*. *whitmani* SGS developed humoral and cellular immune responses to salivary antigens and that this response protects against experimental *L*. *braziliensis* challenge, we investigated the anti-SGS IgG response in individuals naturally exposed to *Lu*. *whitmani*. First, we evaluated antibodies levels in residents (n = 264) from Corte de Pedra, an endemic area where CL is caused by *L*. *braziliensis*, and in subjects (n = 13) from a non-endemic area (controls). Endemic area residents displayed a higher anti-SGS IgG response than controls (Fig 6A) and 104/264 (39,3%) individuals were seropositive. Next, we compared the anti-SGS response in patients with active CL lesions vs. individuals with subclinical *L*. *braziliensis* infection. These are characterized as healthy individuals presenting a positive *Leishmania* skin test (LST) and lack of CL history [22]. We also included endemic area subjects with a negative LST. Importantly, all individuals tested (CL patients, LST+ and LST-) reside in Corte de Pedra and are therefore naturally exposed to *Lu*. *whitmani*, although a variation in exposure intensity can occur according to other demographic indicators such as age, for example. We observed a positive anti- saliva IgG response in all three groups: however, CL patients presented lower (p<0.05) IgG levels compared to LST+ and LST- individuals (Fig 6B). These results suggest that the presence of a humoral response to *Lu*. *whitmani* SGS may occur in parallel to the presence of a cellular immune response to *Leishmania*, as defined by the presence of a positive LST.

## Discussion

Sand fly saliva has immunomodulatory as well as immunogenic proteins and immunity to certain salivary components can confer protection against leishmaniasis (rev. in [11,23]). In the present study, we demonstrate for the first time that immunization with *Lu*. *whitmani* saliva, a vector of *L*. *braziliensis*, generates a specific immune response resulting in long-term protection against infection.

Initially we characterized the immune response to immunization with *Lu*. *whitmani* SGS: mice presented an increased production of anti-*Lu*. *whitmani* SGS antibodies corroborating earlier data regarding the immunogenic properties of sand fly salivary molecules [9]. The antibodies developed by immunized mice recognized distinct salivary proteins (~45kDa,~36kDa, ~30kDaand ~28kDa). Further studies are needed to identify and characterize these molecules, an important aspect since identification of discrete salivary molecules recognized by naturally exposed individuals can contribute to the development of tools to monitor exposure to sand flies [24,25].

Mice immunized with *Lu*. *whitmani* SGS also developed a cellular response as evidenced by the presence of an intense lymphocytic infiltration, characteristic of a delayed-type hypersensitivity response (DTH) which was detected following a challenge inoculation of SGS. Moreover, we also detected production of IFN-γ, IL-10 and IL-4 by draining lymph node cells following stimulation with *Lu*. *whitmani* SGS, as well as the presence of CD4^+^IFN-γ^+^ and CD4^+^IL-10^+^ T cells. Following challenge with *L*. *braziliensis* + *Lu*. *whitmani* SGS, immune mice failed to develop cutaneous lesions and displayed a significantly reduced parasite load at the challenge site. This outcome was paralleled by a significant increase in IFN-γ production in response to stimulation with *Leishmania* antigens. The current hypothesis is that immunization with sand fly salivary molecules primes a CD4^+^ Th1 response, including IFN-γ secreting cells, and this response activates macrophages, promoting *Leishmania* killing [8,11]. Our results are in agreement with this hypothesis although we cannot presently exclude that IFN-γ produced by other cell sources such as NK cells may also be at play. Additionally, we suggest that the IL-10 response detected in mice immunized with *Lu*. *whitmani* SGS may have contributed in regulating the cellular response thus inhibiting lesion development. In *L*. *major* infection, IL-10 is important to prevent cell-mediated immunopathology [26]. The IL-10 response may also have contributed to parasite persistence in draining lymph nodes, which did not differ comparing SGS-immunized vs. control mice. In mice infected with *L*. *braziliensis*, we showed that Tregs secreting IL-10 are observed [27] in accordance with other works that implicated Tregs and IL-10 in *Leishmania* persistence [28,29].

Individuals naturally exposed to sand flies in areas endemic for leishmaniasis develop a humoral immune response to salivary molecules [30].

Herein, we confirmed that individuals residing in a CL endemic area display positive serology to *Lu*. *whitmani* SGS. Moreover, anti-saliva antibody levels were higher in CL patients compared to subclinical individuals, that is those that have a positive LST test without history of CL. Herein, subclinical individuals were characterized as described by Schnorr et al. [18]; however, we are aware that consensus criteria for determining subclinical or asymptomatic infection are lacking [31]. We reported similar results in a Visceral Leishmaniasis endemic area: individuals presenting a positive LST display a higher humoral response to *Lu*. *longipalpis* saliva [32,33] suggesting that induction of an immune response to salivary molecules can facilitate induction of anti-*Leishmania* responses. Our results differ from those obtained in CL areas endemic for *L*. *tropica* [34] and *L*. *major* [35,36]: in these works, CL patients displayed higher antibody titers to *P*. *sergenti* and *P*. *papatasi*, respectively. We suggest that these discrepancies maybe related to differences in salivary components among these species [37].

In terms of human exposure, the results we describe for *Lu*. *whitmani* also differ from those we obtained with *Lu*. *intermedia*: CL patients presented a higher IgG response to *Lu*. *intermedia* SGS compared to individuals with a positive LST [13] and exposure to *Lu*. *intermedia* saliva increases the risk of developing CL caused by *L*. *braziliensis* [15], an effect associated with an IL-10-dominant immune response to salivary antigens from *Lu*. *intermedia*. Experimental immunization with *Lu*. *intermedia* SGS enhanced *L*. *braziliensis* infection [13,14] whereas immunization with *Lu*. *whitmani* protected against CL development (herein). Thus, it appears that even for sand flies belonging to the same subgenus (Nyssomyia) such as *Lu*. *intermedia* and *Lu*. *whitmani*, exposure to salivary molecules leads to distinct outcomes and this is not attributed to differences in the experimental procedure as in all these works, we employed the same immunization scheme and intradermal challenge. One important aspect that has not been addressed, so far, is whether these outcomes are also obtained following a challenge with *L*. *braziliensis*-infected *Lu*. *intermedia* or *Lu*. *whitmani* sand flies. This is relevant because it has been shown that the complex scenario present in a live sand fly challenge is not fully mimicked by needle challenge [38,39]. Regarding differences in the antigenicity of salivary proteins, the sialome of *Lu*. *intermedia* has been described [40] but characterization of the salivary proteins from *Lu*. *whitmani* is still lacking so we cannot, presently, probe for possible differences that could explain the distinct outcomes.

Although *Lu*. *whitmani* and *Lu*. *intermedia* can be found in the same CL endemic areas we do not know if there is a cross-protective immune response to these two species of sand flies. Previous work has shown that immunization with saliva from closely related species of sand flies could result in cross-protection. Lestinova *et al* demonstrated that immunization with *P*. *papatasi* saliva resulted in protection upon infection with *L*. *major* plus *P*. *papatasi* or *P*. *duboscqi* saliva [41]. However, mice immunized with *Lu*. *longipalpis* saliva were protected against *L*. *amazonensis* only against needle injection of a homologous sand fly saliva challenge; protection was not observed when mice were challenged with *Phlebotomus sp*. saliva [42] highlighting this aspect of sand fly specificity. In our hands, immunization with *Lu*. *longipalpis* saliva protected against a challenge with *L*. *braziliensis* plus *Lu*. *intermedia* saliva [43], indicating that common antigens may exist across different species. Collectively, these studies highlight differences in salivary components [37], as mentioned before. They also reinforce the need to invest in characterizing salivary components and to perform systematic experiments to identify cross protective antigens.

Additionally, other components present in the infected sand fly, such as the promastigote secretory gel (PSG) and parasite exosomes, are also inoculated alongside parasites and saliva into the host skin [44]. These components could play important roles in exposure to sand flies and highlight the importance of testing vaccine candidates against the challenge of infected sand fly bites [38]. Together, our data reinforce the possibility of employing sand fly salivary molecules as components for a leishmaniasis vaccine [9,10,40,45]. In parallel, we also envisage the development of *Lu*. *whitmani* salivary molecules as tools to monitor human exposure in CL endemic areas [46,47].

## Supporting Information

S1 Checklist STROBE Checklist (DOCX)
