# Supplementary material for: Immunity to Lutzomyia whitmani Saliva Protects against Experimental Leishmania braziliensis Infection
Source: PLoS Negl Trop Dis. 2016 Nov 3;10(11):e0005078. doi: 10.1371/journal.pntd.0005078 (PMC5094744; doi:10.1371/journal.pntd.0005078)
Supplement: S1 Checklist — (DOCX) [file pntd.0005078.s001.docx]

STROBE Statement—checklist of items that should be included in reports of observational studies

**YOU MUST NOTE THE PAGE NUMBER WHERE EACH ITEM IS REPORTED INSIDE THE BRACKETS [ ]. IF NOT APPLICABLE WRITE N/A**

|  | **Item No** | **Recommendation** |
| --- | --- | --- |
| **Title and abstract** | 1 | (*a*) Indicate the study’s design with a commonly used term in the title or the |
|  |  | abstract **[Within the title page 1]** |
|  |  | (*b*) Provide in the abstract an informative and balanced summary of what was |
|  |  | done and what was found **[See methods and findings section of abstract page 2 ]** |
| **Introduction** |  |  |
| Background/rationale | 2 | Explain the scientific background and rationale for the investigation being |
|  |  | reported **[ page 4 ]** |
| Objectives | 3 | State specific objectives, including any prespecified hypotheses **[pages 4 ]** |
| **Methods** |  |  |
| Study design | 4 | Present key elements of study design early in the paper **[ Methods page 4 ]** |
| Setting | 5 | Describe the setting, locations, and relevant dates, including periods of |
|  |  | recruitment, exposure, follow-up, and data collection **[N/A ]** |
| Participants | 6 | (*a*) *Cohort study*—Give the eligibility criteria, and the sources and methods of |
|  |  | selection of participants. Describe methods of follow-up **[page 9 ]** |
|  |  | *Case-control study*—Give the eligibility criteria, and the sources and methods of |
|  |  | case ascertainment and control selection. Give the rationale for the choice of cases |
|  |  | and controls **[ ]** |
|  |  | *Cross-sectional study*—Give the eligibility criteria, and the sources and methods |
|  |  | of selection of participants **[ ]** |
|  |  | (*b*) *Cohort study*—For matched studies, give matching criteria and number of |
|  |  | exposed and unexposed **[ ]** |
|  |  | *Case-control study*—For matched studies, give matching criteria and the number |
|  |  | of controls per case **[N/A ]** |
| Variables | 7 | Clearly define all outcomes, exposures, predictors, potential confounders, and |
|  |  | effect modifiers. Give diagnostic criteria, if applicable **[N/A]** |
| Data sources/ measurement | 8* | For each variable of interest, give sources of data and details of methods of |
|  |  | assessment (measurement). Describe comparability of assessment methods if there |
|  |  | is more than one group **[ Methods pages 6-10 ]** |
| Bias | 9 | Describe any efforts to address potential sources of bias **[N/A ]** |
| Study size | 10 | Explain how the study size was arrived at **[N/A ]** |
| Quantitative variables | 11 | Explain how quantitative variables were handled in the analyses. If applicable, |
|  |  | describe which groupings were chosen and why **[N/A]** |
| Statistical methods | 12 | (*a*) Describe all statistical methods, including those used to control for |
|  |  | confounding **[ Methods page 10 ]** |
|  |  | (*b*) Describe any methods used to examine subgroups and interactions **[ N/A]** |

(*c*) Explain how missing data were addressed **[N/A ]**

(*d*) *Cohort study*—If applicable, explain how loss to follow-up was addressed **[ N/A ]** *Case-control study*—If applicable, explain how matching of cases and controls was addressed **[ ]**

*Cross-sectional study*—If applicable, describe analytical methods taking account of sampling strategy **[ N/A ]**

(*e*) Describe any sensitivity analyses **[N/A ]**

Continued on next page

1

**Results**

| Participants | 13* | (a) Report numbers of individuals at each stage of study—eg numbers potentially eligible, | |
| --- | --- | --- | --- |
|  |  | examined for eligibility, confirmed eligible, included in the study, completing follow-up, and | |
|  |  | analysed **[ page 13 ]** |  |
|  |  | (b) Give reasons for non-participation at each stage **[ N/A ]** |  |
|  |  | (c) Consider use of a flow diagram **[N/A ]** |  |
| Descriptive | 14* | (a) Give characteristics of study participants (eg demographic, clinical, social) and information | |
| data |  | on exposures and potential confounders **[ N/A]** |  |
|  |  | (b) Indicate number of participants with missing data for each variable of interest | **[N/A]** |
|  |  | (c) *Cohort study*—Summarise follow-up time (eg, average and total amount) **[N/A** | **]** |
| Outcome data | 15* | *Cohort study*—Report numbers of outcome events or summary measures over time **[ ]** | |
|  |  | *Case-control study—*Report numbers in each exposure category, or summary measures of | |
|  |  | exposure **[ ]** |  |
|  |  | *Cross-sectional study—*Report numbers of outcome events or summary measures **[N/A ]** | |
| Main results | 16 | (*a*) Give unadjusted estimates and, if applicable, confounder-adjusted estimates and their | |
|  |  | precision (eg, 95% confidence interval). Make clear which confounders were adjusted for and | |
|  |  | why they were included **[N/A ]** |  |
|  |  | (*b*) Report category boundaries when continuous variables were categorized **[N/A ]** | |
|  |  | (*c*) If relevant, consider translating estimates of relative risk into absolute risk for a meaningful | |
|  |  | time period **[ N/A ]** |  |
| Other analyses | 17 | Report other analyses done—eg analyses of subgroups and interactions, and sensitivity | |
|  |  | analyses **[ N/A]** |  |
| **Discussion** |  |  |  |
| Key results | 18 | Summarise key results with reference to study objectives **[ page 14 ]** |  |
| Limitations | 19 | Discuss limitations of the study, taking into account sources of potential bias or imprecision. | |
|  |  | Discuss both direction and magnitude of any potential bias **[ N/A ]** |  |
| Interpretation | 20 | Give a cautious overall interpretation of results considering objectives, limitations, multiplicity | |
|  |  | of analyses, results from similar studies, and other relevant evidence **[ page 14-18 ]** | |
| Generalisability | 21 | Discuss the generalisability (external validity) of the study results **[N/A ]** | |
| **Other information** | |  |  |
| Funding | 22 | Give the source of funding and the role of the funders for the present study and, if applicable, | |
|  |  | for the original study on which the present article is based **[this is supplied in the PLoS online submission system ]** | |

*Give information separately for cases and controls in case-control studies and, if applicable, for exposed and unexposed groups in cohort and cross-sectional studies.

**Note:** An Explanation and Elaboration article discusses each checklist item and gives methodological background and published examples of transparent reporting. The STROBE checklist is best used in conjunction with this article (freely available on the Web sites of PLoS Medicine at http://www.plosmedicine.org/, Annals of Internal Medicine at http://www.annals.org/, and Epidemiology at http://www.epidem.com/). Information on the STROBE Initiative is available at www.strobe-statement.org.

**Once you have completed this checklist, please save a copy and upload it as part of your submission. When requested to do so as part of the upload process, please select the file type: *Checklist*. You will NOT be able to proceed with submission unless the checklist has been uploaded. Please DO NOT include this checklist as part of the main manuscript document. It must be uploaded as a separate file.**

2
